# 7-Deacetyl­gedunin

**DOI:** 10.1107/S1600536810052037

**Published:** 2010-12-18

**Authors:** Warin Ravangpai, Thapong Theerawattananond, Somjai Pengpreecha, Nongnuj Muangsin, Khanitha Pudhom

**Affiliations:** a Research Centre of Bioorganic Chemistry, Department of Chemistry, Faculty of Science, Chulalongkorn University, Bangkok 10330, Thailand; b Research Centre of Bioorganic Chemistry and Center for Petroleum, Petrochemicals and Advanced Materials, Chulalongkorn University, Bangkok 10330, Thailand

## Abstract

The title compound [systematic name: (1*S*,3a*S*,4a*R*,4b*S*,5*R*,6a*R*,10a*R*,10b*R*,12a*S*)-1-(furan-3-yl)-5-hy­droxy-4b,7,7,10a,12a-penta­methyl-4b,5,6,6a,7,10a,10b,11,12,12a-deca­hydro­naphtho­[2,1-*f*]oxireno[2,3-*d*]isochromene-3,8(1*H*,3a*H*)-dione], C_26_H_32_O_6_, which is a limonoid-type triterpene isolated from the seeds of *X. moluccensis*, crystallizes with three independent mol­ecules with very similar geometries in the asymmetric unit. In each mol­ecule, the four fused six-membered rings of the genudin core adopt distorted half-chair, chair, twist-boat and twisted half-chair conformations. In the crystal, inter­molecular O—H⋯O hydrogen bonds link the mol­ecules into helical chains propagated in [100]. Weak non-classical C—H⋯O contacts further consolidate the crystal packing.

## Related literature

For general background to limonoid and its potential bio­activity, see: Koul *et al.* (2004 ▶); Endo *et al.* (2002 ▶); Nakagawa *et al.* (2001 ▶); Pudhom *et al.* (2009 ▶). For related structures, see: Waratchareeyakul *et al.* (2004 ▶); Hofer *et al.* (2009 ▶). For limonoids with novel skeletons extracted from *Xylocarpus granatum* Koenig, see: Wu *et al.* (2004 ▶); Cui *et al.* (2005 ▶, 2007 ▶); Zhou *et al.* (2006 ▶); Li *et al.* (2009 ▶). The absolute configuration was assigned on the basis of literature data, see: Pudhom *et al.* (2009 ▶); Mitsui *et al.* (2006); ▶ Waratchareeyakul *et al.* (2004 ▶); Hofer *et al.* (2009 ▶).
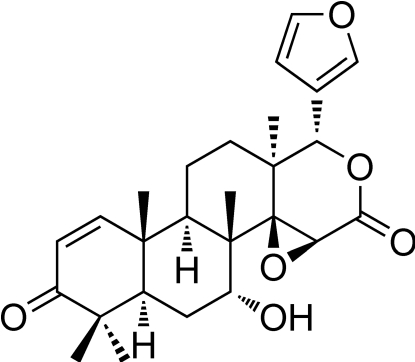


## Experimental

### 

#### Crystal data

C_26_H_32_O_6_
*M* *_r_* = 440.52
Orthorhombic, 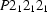

*a* = 12.3642 (4) Å
*b* = 12.8445 (5) Å
*c* = 43.5584 (15) Å
*V* = 6917.6 (4) Å^3^
*Z* = 12
Mo *K*α radiation
μ = 0.09 mm^−1^
*T* = 296 K
0.48 × 0.40 × 0.20 mm

#### Data collection

Bruker SMART APEXII CCD area-detector diffractometer
38709 measured reflections
9382 independent reflections
6530 reflections with *I* > 2σ(*I*)
*R*_int_ = 0.059

#### Refinement

*R*[*F* ^2^ > 2σ(*F* ^2^)] = 0.068
*wR*(*F* ^2^) = 0.212
*S* = 0.99
9382 reflections
891 parameters
H atoms treated by a mixture of independent and constrained refinement
Δρ_max_ = 0.41 e Å^−3^
Δρ_min_ = −0.40 e Å^−3^

### 

Data collection: *APEX2* (Bruker, 2008 ▶); cell refinement: *SAINT* (Bruker, 2008 ▶); data reduction: *SAINT*; program(s) used to solve structure: *SHELXS97* (Sheldrick, 2008 ▶); program(s) used to refine structure: *SHELXL97* (Sheldrick, 2008 ▶); molecular graphics: *ORTEP-3* (Farrugia, 1997 ▶); software used to prepare material for publication: *publCIF* (Westrip, 2010 ▶).

## Supplementary Material

Crystal structure: contains datablocks global, I. DOI: 10.1107/S1600536810052037/cv5013sup1.cif

Structure factors: contains datablocks I. DOI: 10.1107/S1600536810052037/cv5013Isup2.hkl

Additional supplementary materials:  crystallographic information; 3D view; checkCIF report

## Figures and Tables

**Table 1:** Hydrogen-bond geometry (Å, °)

*D*—H⋯*A*	*D*—H	H⋯*A*	*D*⋯*A*	*D*—H⋯*A*
O4*A*—H4*OA*⋯O1	1.01 (6)	1.83 (6)	2.818 (5)	169 (5)
O4—H4*O*⋯O1*B*	0.81 (5)	2.14 (5)	2.952 (5)	177 (4)
O4*B*—H4*OB*⋯O5*A*^i^	0.88 (7)	2.06 (7)	2.849 (5)	149 (6)
C7—H7⋯O1*A*	0.98	2.55	3.525 (6)	172
C11—H11*A*⋯O3^ii^	0.97	2.45	3.278 (6)	143
C21*B*—H21*B*⋯O5*B*^iii^	0.93	2.52	3.369 (9)	151

Symmetry codes: (i) 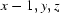; (ii) 
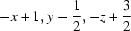
; (iii) 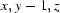.

**Fig. 1.**
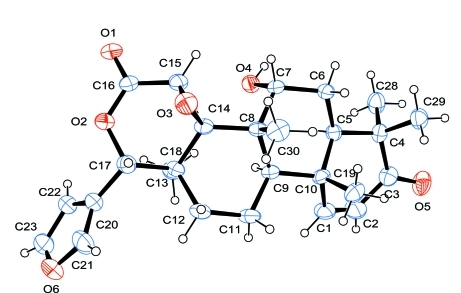
The molecular structure of the title compound showing 30% probability displacement ellipsoids and the atom-numbering scheme in one independent molecule.

## supplementary crystallographic information

### Comment

Limonoid research from the Meliaceae family is of growing interest due to a range of biological activities, such as insect antifeedants and growth regulators, antibacterial, antifungal, antimalarial, anticancer and antiviral activities on humans (Koul *et al.*, 2004; Endo *et al.*, 2002; Nakagawa *et al.*, 2001). Such a focused interest upon limonoids from the family Meliaceae has already resulted in the discovery of several limonoids with novel skeletons, mostly, but not exclusively, from within the genus *Xylocarpus*, and, in particular, the cannonball mangrove, *Xylocarpus granatum* Koenig (Wu *et al.*, 2004; Cui *et al.*, 2005; Zhou *et al.*, 2006*;* Cui *et al.*, 2007*;* Li *et al.*, 2009*;* Pudhom *et al.*, 2009). Limonoid derivatives have been found in all *Xylocarpus* plants studied, but their distribution and content may vary both between different plant species, and between parts or geocultivars of the same species. This, combined with their wide ranging structural diversity and potential biological significance across this plant family, prompted us to investigate another plant in this genus, *Xylocarpus moluccensis*. Herein, the complete assignments of NMR data and the crystal and molecular structure of 7-deacetylgedunin obtained from the seeds of *X. moluccensis*, were reported for the first time.

The asymmetric unit of the title compound contains three crystallographically independent molecules. There is very little difference between the bond lengths and angles of these molecules. The molecule consists of four fused six membered rings (A, B, C and D) of genudin core. All the rings are all *trans* fused and adopt distorted half-chair, chair, twist-boat, and twisted half-chair conformation, respectively. The furan ring is planar and nearly perpendicular orientation to the main plane of the molecule.

In the crystal structure, molecules related by translation along the *a* axis are linked into chains through O—H···O hydrogen bonds. Weak non-classical C—H···O contacts are also observed in the structure.

The absolute configuration was assigned on the basis of literature data (Pudhom *et al.*, 2009; Mitsui *et al.*, 2006; Waratchareeyakul *et al.*, 2004; Hofer *et al.*, 2009).

### Experimental

General Experimental Procedures. Melting point was measured using a Fisher-Johns melting point apparatus. HRESIMS spectrum was obtained using a Bruker micrOTOF mass spectrometer. The NMR spectra were recorded on a Varian YH400 spectrometer at 400 MHz for ^1^H NMR and at 100 MHz for ^13^C NMR using TMS (tetramethylsilane) as internal standard.

Plant Material. Fruits of *X. moluccensis* were collected from Phuket Province, Thailand, in December 2009. Plants materials were identified by Royal Forest Department, Bangkok, Thailand.

Extraction and Isolation of 7-Deacetylgedunin (1). Air-dried powdered seeds of *X. moluccensis* (3 kg) were extracted with MeOH (5*L x* 2, each for three days). The extract was concentrated under reduced pressure, followed by suspension in water and extraction with EtOAc. The resulting EtOAc crude extract (35 g) was chromatographed on a silica gel column eluted with a gradient of acetone–n-hexane (from 1:9 to 1:0) to yield 12 fractions. Fraction 7 (2.80 g) was then applied to a silica gel column and eluted with a 1:2 mixture of acetone–n-hexane to afford 12 subfractions. Subfraction 4 was further purified by silica gel column chromatography (acetone–n-hexane; 1:4) and recrystallized from EtOAc–n-hexane (1:1) to afford 7-deacetylgedunin (1, 15.0 mg).

7-Deacetylgedunin (1): colorless crystals; mp 246–247 *^o^*C; ^1^H NMR (400 MHz, CDCl_3_) d 7.41 (1*H*, s, H-21), 7.40 (1*H*, s, H-23), 7.10 (1*H*, d, *J* = 10.4 Hz, H-1), 6.35 (1*H*, s, H-22), 5.84 (1*H*, d, *J* = 10.4 Hz, H-2), 5.60 (1*H*, s, H-17), 3.90 (1*H*, s, H-15), 3.57 (1*H*, br s, H-7), 2.52 (1*H*, m, H-9), 2.49 (1*H*, m, H-5), 1.95 (1*H*, m, H-11*b*), 1.89 (1*H*, m, H-6a), 1.80 (1*H*, m, H-11*b*), 1.71 (1*H*, m, H-12*a*), 1.69 (1*H*, m, H-6 b), 1.54 (1*H*, m, H-12*b*), 1.23 (3*H*, s, CH_3_-18), 1.19 (3*H*, s, CH_3_-19), 1.14 (3*H*, s, CH_3_-28), 1.09 (3*H*, s, CH_3_-29), 1.00 (3*H*, s, CH_3_-30); ^13^C NMR (100 MHz, CDCl_3_) d 204.6 (C, C-3), 168.3 (C, C-16), 157.8 (CH, C-1), 143.0 (CH, C-23), 141.2 (CH, C-21), 125.7 (CH, C-2), 120.6 (C, C-20), 110.0 (CH, C-22), 78.5 (CH, C-17), 70.0 (C, C-14), 69.7 (CH, C-7), 57.8 (CH, C-15), 44.6 (CH, C-5), 44.2 (C, C-4), 43.7 (C, C-8), 40.7 (C, C-10), 38.3 (C, C-13), 38.0 (CH, C-9), 27.3 (CH_2_, C-6), 27.2 (CH_3_, C-28), 26.4 (CH_2_, C-12), 21.5 (CH_3_, C-29), 19.9 (CH_3_, C-19), 18.7 (CH_3_, C-30), 17.8 (CH_3_, C-18), 15.0 (CH_2_, C-11); HRESIMS *m/z* 463.2101 [*M*+Na]^+^ (calcd for C_26_H_32_O_6_Na, 463.2097).

### Refinement

C-bound H atoms were geometrically positioned and treated as riding atoms with distances C—H = 0.96 Å (CH_3_), 0.97 Å (CH_2_), 0.93 Å (CH), and *U*_iso_(H) = 1.20 *U*_eq_(C) for methylene and aromatic, 1.50 *U*_eq_(C) for methyl. O-bound H atoms were located on a difference map and refined isotropically. The absolute structure could not be determined from the X-ray analysis, but it was known from earlier work on related compounds (*e.g.* Pudhom *et al.*, 2009; Mitsui *et al.*, 2006; Waratchareeyakul *et al.*, 2004). 6,498 Friedel pairs were therefore merged before the final refinement.

### Crystal data

**Table:**

C_26_H_32_O_6_	*Z* = 12
*M**_r_* = 440.52	*F*(000) = 2832
Orthorhombic, *P*2_1_2_1_2_1_	*D*_x_ = 1.269 Mg m^−^^3^
Hall symbol: P 2ac 2ab	Mo *K*α radiation, λ = 0.71073 Å
*a* = 12.3642 (4) Å	µ = 0.09 mm^−^^1^
*b* = 12.8445 (5) Å	*T* = 296 K
*c* = 43.5584 (15) Å	Prism, colourless
*V* = 6917.6 (4) Å^3^	0.48 × 0.40 × 0.20 mm

### Data collection

**Table:**

Bruker SMART APEXII CCD area-detector diffractometer	6530 reflections with *I* > 2σ(*I*)
Radiation source: Mo Kα	*R*_int_ = 0.059
graphite	θ_max_ = 28.3°, θ_min_ = 1.7°
φ and ω scans	*h* = −8→16
38709 measured reflections	*k* = −17→17
9382 independent reflections	*l* = −28→58

### Refinement

**Table:**

Refinement on *F*^2^	H atoms treated by a mixture of independent and constrained refinement
Least-squares matrix: full	*w* = 1/[σ^2^(*F*_o_^2^) + (0.1415*P*)^2^ + 0.6215*P*] where *P* = (*F*_o_^2^ + 2*F*_c_^2^)/3
*R*[*F*^2^ > 2σ(*F*^2^)] = 0.068	(Δ/σ)_max_ = 0.011
*wR*(*F*^2^) = 0.212	Δρ_max_ = 0.41 e Å^−^^3^
*S* = 0.99	Δρ_min_ = −0.40 e Å^−^^3^
9382 reflections	Extinction correction: *SHELXL97* (Sheldrick, 2008), Fc^*^=kFc[1+0.001xFc^2^λ^3^/sin(2θ)]^-1/4^
891 parameters	Extinction coefficient: 0.0120 (11)
0 restraints	

### Special details

**Table:**

Geometry. All e.s.d.'s (except the e.s.d. in the dihedral angle between two l.s. planes) are estimated using the full covariance matrix. The cell e.s.d.'s are taken into account individually in the estimation of e.s.d.'s in distances, angles and torsion angles; correlations between e.s.d.'s in cell parameters are only used when they are defined by crystal symmetry. An approximate (isotropic) treatment of cell e.s.d.'s is used for estimating e.s.d.'s involving l.s. planes.
Refinement. Refinement of *F*^2^ against ALL reflections. The weighted *R*-factor *wR* and goodness of fit *S* are based on *F*^2^, conventional *R*-factors *R* are based on *F*, with *F* set to zero for negative *F*^2^. The threshold expression of *F*^2^ > σ(*F*^2^) is used only for calculating *R*-factors(gt) *etc*. and is not relevant to the choice of reflections for refinement. *R*-factors based on *F*^2^ are statistically about twice as large as those based on *F*, and *R*- factors based on ALL data will be even larger.

### Fractional atomic coordinates and isotropic or equivalent isotropic displacement parameters (Å^2^)

**Table:**

	*x*	*y*	*z*	*U*_iso_*/*U*_eq_	
C1	0.2329 (5)	0.3351 (4)	0.79409 (14)	0.0681 (15)	
H1	0.2783	0.2887	0.784	0.082*	
C2	0.1516 (6)	0.2994 (4)	0.81028 (17)	0.0771 (17)	
H2	0.1417	0.2277	0.8114	0.092*	
C3	0.0755 (5)	0.3666 (4)	0.82667 (12)	0.0584 (12)	
C4	0.1132 (4)	0.4728 (4)	0.83638 (10)	0.0506 (11)	
C5	0.2300 (4)	0.4960 (3)	0.82397 (9)	0.0428 (9)	
H5	0.279	0.4592	0.838	0.051*	
C6	0.2623 (4)	0.6099 (3)	0.82612 (10)	0.0452 (9)	
H6A	0.2441	0.6363	0.8463	0.054*	
H6B	0.2219	0.6498	0.8111	0.054*	
C7	0.3837 (4)	0.6246 (3)	0.82045 (9)	0.0442 (9)	
H7	0.4011	0.6989	0.8217	0.053*	
C8	0.4174 (4)	0.5835 (3)	0.78881 (9)	0.0399 (9)	
C9	0.3780 (4)	0.4687 (3)	0.78542 (9)	0.0406 (9)	
H9	0.4156	0.4304	0.8017	0.049*	
C10	0.2539 (4)	0.4503 (3)	0.79171 (10)	0.0447 (10)	
C11	0.4186 (5)	0.4204 (4)	0.75536 (10)	0.0572 (12)	
H11A	0.4168	0.3452	0.7574	0.069*	
H11B	0.3687	0.4391	0.7391	0.069*	
C12	0.5328 (4)	0.4528 (4)	0.74565 (9)	0.0493 (11)	
H12A	0.5275	0.5078	0.7305	0.059*	
H12B	0.5683	0.3939	0.7361	0.059*	
C13	0.6023 (4)	0.4911 (3)	0.77253 (9)	0.0418 (9)	
C14	0.5430 (4)	0.5869 (3)	0.78525 (9)	0.0416 (9)	
C15	0.6123 (4)	0.6585 (4)	0.80297 (12)	0.0543 (11)	
H15	0.5781	0.6984	0.8195	0.065*	
C16	0.7301 (4)	0.6339 (4)	0.80624 (12)	0.0576 (12)	
C17	0.7122 (4)	0.5284 (4)	0.76025 (10)	0.0522 (11)	
H17	0.6994	0.5809	0.7443	0.063*	
C18	0.6207 (4)	0.4083 (4)	0.79754 (10)	0.0511 (11)	
H18A	0.6939	0.4128	0.8048	0.077*	
H18B	0.6081	0.3403	0.7891	0.077*	
H18C	0.5717	0.4203	0.8143	0.077*	
C19	0.1780 (5)	0.4887 (5)	0.76595 (11)	0.0638 (14)	
H19A	0.2011	0.4602	0.7467	0.096*	
H19B	0.1804	0.5634	0.765	0.096*	
H19C	0.1054	0.4666	0.7702	0.096*	
C20	0.7835 (4)	0.4456 (5)	0.74728 (12)	0.0619 (14)	
C21	0.7901 (5)	0.4192 (6)	0.71699 (13)	0.0825 (19)	
H21	0.7509	0.4507	0.7013	0.099*	
C22	0.8579 (5)	0.3800 (5)	0.76247 (14)	0.0716 (16)	
H22	0.8724	0.3793	0.7834	0.086*	
C23	0.9039 (5)	0.3185 (6)	0.7410 (2)	0.089 (2)	
H23	0.9566	0.2686	0.745	0.107*	
C28	0.1188 (5)	0.4697 (6)	0.87202 (12)	0.0689 (15)	
H28A	0.1419	0.5362	0.8796	0.103*	
H28B	0.1694	0.4172	0.8783	0.103*	
H28C	0.0486	0.4536	0.8801	0.103*	
C29	0.0279 (4)	0.5528 (4)	0.82682 (13)	0.0620 (13)	
H29A	0.0509	0.6211	0.833	0.093*	
H29B	−0.0397	0.5365	0.8365	0.093*	
H29C	0.0192	0.5512	0.8049	0.093*	
C30	0.3711 (5)	0.6552 (4)	0.76355 (11)	0.0583 (12)	
H30A	0.3921	0.6293	0.7438	0.087*	
H30B	0.3988	0.7244	0.7662	0.087*	
H30C	0.2936	0.6564	0.765	0.087*	
O1	0.7827 (3)	0.6660 (3)	0.82751 (10)	0.0790 (12)	
O2	0.7768 (3)	0.5761 (3)	0.78487 (8)	0.0626 (9)	
O3	0.5820 (3)	0.6830 (3)	0.77226 (9)	0.0639 (9)	
O4	0.4456 (3)	0.5692 (3)	0.84286 (7)	0.0515 (8)	
H4O	0.417 (4)	0.585 (4)	0.8589 (11)	0.036 (11)*	
O5	−0.0150 (3)	0.3360 (4)	0.83266 (11)	0.0803 (12)	
O6	0.8623 (4)	0.3401 (5)	0.71286 (12)	0.0992 (16)	
C1A	0.9781 (4)	1.0580 (5)	0.92122 (11)	0.0641 (15)	
H1A	0.9379	1.1107	0.9304	0.077*	
C2A	1.0459 (5)	1.0028 (5)	0.93859 (11)	0.0684 (16)	
H2A	1.0496	1.017	0.9595	0.082*	
C3A	1.1140 (4)	0.9214 (4)	0.92606 (10)	0.0503 (10)	
C4A	1.0875 (4)	0.8730 (4)	0.89477 (10)	0.0484 (10)	
C5A	0.9809 (4)	0.9208 (3)	0.88216 (8)	0.0419 (9)	
H5A	0.9239	0.8878	0.8944	0.05*	
C6A	0.9539 (4)	0.8910 (4)	0.84898 (9)	0.0518 (11)	
H6A1	0.9997	0.9301	0.8351	0.062*	
H6A2	0.9684	0.8175	0.8459	0.062*	
C7A	0.8359 (4)	0.9135 (3)	0.84180 (8)	0.0436 (10)	
H7A	0.8217	0.894	0.8204	0.052*	
C8A	0.8068 (4)	1.0282 (3)	0.84607 (8)	0.0404 (9)	
C9A	0.8434 (4)	1.0640 (3)	0.87876 (9)	0.0400 (9)	
H9A	0.794 (2)	1.017 (2)	0.8948 (7)	0.048*	
C10A	0.9641 (4)	1.0386 (3)	0.88735 (9)	0.0437 (9)	
C11A	0.8101 (4)	1.1775 (4)	0.88503 (13)	0.0551 (12)	
H11C	0.8621	1.2234	0.8753	0.066*	
H11D	0.8134	1.19	0.907	0.066*	
C12A	0.6951 (4)	1.2064 (4)	0.87344 (13)	0.0535 (12)	
H12C	0.6599	1.25	0.8886	0.064*	
H12D	0.7014	1.2464	0.8546	0.064*	
C13A	0.6247 (4)	1.1109 (3)	0.86738 (9)	0.0439 (9)	
C14A	0.6823 (4)	1.0445 (3)	0.84370 (8)	0.0432 (9)	
C15A	0.6110 (4)	0.9696 (4)	0.82740 (10)	0.0546 (12)	
H15A	0.6435	0.9043	0.8203	0.066*	
C16A	0.4943 (4)	0.9683 (4)	0.83523 (10)	0.0568 (12)	
C17A	0.5149 (4)	1.1464 (4)	0.85369 (12)	0.0580 (12)	
H17A	0.5292	1.1868	0.835	0.07*	
C18A	0.5998 (4)	1.0475 (4)	0.89699 (9)	0.0497 (10)	
H18D	0.5233	1.036	0.8985	0.075*	
H18E	0.6244	1.0856	0.9146	0.075*	
H18F	0.6365	0.9816	0.8961	0.075*	
C19A	1.0486 (4)	1.1073 (4)	0.87135 (16)	0.0673 (14)	
H19D	1.0236	1.178	0.8709	0.101*	
H19E	1.0595	1.083	0.8507	0.101*	
H19F	1.1157	1.1038	0.8824	0.101*	
C20A	0.4448 (4)	1.2091 (4)	0.87418 (14)	0.0638 (14)	
C21A	0.4463 (6)	1.3170 (5)	0.87942 (19)	0.087 (2)	
H21A	0.4932	1.3637	0.87	0.105*	
C23A	0.3716 (5)	1.3422 (5)	0.8996 (2)	0.084 (2)	
H23A	0.3582	1.4089	0.907	0.101*	
C22A	0.3633 (6)	1.1741 (6)	0.8930 (2)	0.098 (2)	
H22A	0.3425	1.1049	0.8952	0.118*	
C28A	1.1867 (5)	0.8851 (6)	0.87352 (14)	0.0790 (18)	
H28D	1.1988	0.9575	0.8694	0.118*	
H28E	1.1736	0.849	0.8546	0.118*	
H28F	1.2493	0.8561	0.8834	0.118*	
C29A	1.0675 (5)	0.7570 (4)	0.90139 (12)	0.0665 (14)	
H29D	1.1319	0.7265	0.9098	0.1*	
H29E	1.0488	0.7219	0.8827	0.1*	
H29F	1.0093	0.75	0.9158	0.1*	
C30A	0.8591 (5)	1.0937 (4)	0.82029 (11)	0.0625 (13)	
H30D	0.9363	1.0858	0.821	0.094*	
H30E	0.8407	1.1657	0.8231	0.094*	
H30F	0.8329	1.0704	0.8007	0.094*	
O1A	0.4394 (4)	0.8906 (3)	0.83252 (10)	0.0811 (12)	
O2A	0.4484 (3)	1.0553 (3)	0.84539 (9)	0.0640 (9)	
O3A	0.6444 (3)	1.0643 (3)	0.81249 (7)	0.0626 (10)	
O4A	0.7686 (3)	0.8530 (3)	0.86112 (7)	0.0523 (8)	
H4OA	0.777 (5)	0.782 (5)	0.8515 (13)	0.065 (16)*	
O5A	1.1935 (3)	0.8921 (4)	0.94055 (9)	0.0722 (11)	
O6A	0.3180 (4)	1.2554 (5)	0.90785 (18)	0.120 (2)	
C1B	0.7433 (4)	1.0404 (5)	0.97920 (15)	0.0711 (17)	
H1B	0.7981	0.9999	0.9708	0.085*	
C2B	0.7529 (4)	1.1433 (5)	0.97816 (15)	0.0710 (16)	
H2B	0.8131	1.1721	0.9685	0.085*	
C3B	0.6718 (5)	1.2134 (4)	0.99168 (12)	0.0594 (13)	
C4B	0.5592 (4)	1.1725 (4)	0.99750 (11)	0.0520 (11)	
C5B	0.5477 (3)	1.0577 (3)	0.98712 (9)	0.0402 (9)	
H5B	0.5434	1.0615	0.9647	0.048*	
C6B	0.4429 (3)	1.0062 (4)	0.99666 (9)	0.0442 (9)	
H6B1	0.3829	1.053	0.9927	0.053*	
H6B2	0.4447	0.9914	1.0185	0.053*	
C7B	0.4263 (3)	0.9037 (3)	0.97855 (8)	0.0365 (8)	
H7B	0.3584	0.8712	0.985	0.044*	
C8B	0.5194 (3)	0.8268 (3)	0.98340 (8)	0.0347 (8)	
C9B	0.6275 (3)	0.8821 (3)	0.97583 (9)	0.0392 (9)	
H9B	0.6216	0.902	0.9542	0.047*	
C10B	0.6472 (3)	0.9864 (4)	0.99346 (10)	0.0468 (10)	
C11B	0.7244 (3)	0.8066 (4)	0.97729 (12)	0.0528 (12)	
H11E	0.7814	0.8336	0.9642	0.063*	
H11F	0.7519	0.8061	0.9982	0.063*	
C12B	0.7007 (4)	0.6938 (4)	0.96770 (11)	0.0480 (10)	
H12E	0.7648	0.664	0.9583	0.058*	
H12F	0.6835	0.653	0.9858	0.058*	
C13B	0.6063 (3)	0.6881 (3)	0.94497 (8)	0.0350 (8)	
C14B	0.5059 (3)	0.7298 (3)	0.96164 (8)	0.0333 (8)	
C15B	0.4036 (3)	0.7000 (4)	0.94751 (9)	0.0411 (9)	
H15B	0.3432	0.7492	0.9491	0.049*	
C16B	0.4068 (3)	0.6311 (4)	0.91999 (9)	0.0454 (10)	
C17B	0.5830 (3)	0.5723 (3)	0.93720 (9)	0.0386 (8)	
H17B	0.5662	0.5354	0.9563	0.046*	
C18B	0.6283 (4)	0.7494 (4)	0.91519 (9)	0.0459 (10)	
H18G	0.622	0.8226	0.9192	0.069*	
H18H	0.5766	0.7295	0.8998	0.069*	
H18I	0.7	0.7341	0.908	0.069*	
C19B	0.6749 (5)	0.9765 (5)	1.02792 (12)	0.0696 (16)	
H19G	0.7261	0.9211	1.0308	0.104*	
H19H	0.6103	0.9615	1.0393	0.104*	
H19I	0.7056	1.0407	1.0351	0.104*	
C20B	0.6723 (4)	0.5152 (4)	0.92109 (10)	0.0460 (10)	
C21B	0.7413 (5)	0.4496 (4)	0.93466 (14)	0.0652 (14)	
H21B	0.7393	0.4309	0.9553	0.078*	
C22B	0.7025 (5)	0.5200 (4)	0.88938 (13)	0.0629 (13)	
H22B	0.669	0.5584	0.874	0.076*	
C23B	0.7888 (5)	0.4578 (5)	0.88646 (18)	0.0765 (18)	
H23B	0.8256	0.4465	0.8681	0.092*	
C28B	0.4850 (5)	1.2395 (5)	0.97733 (15)	0.0708 (15)	
H28G	0.4947	1.3116	0.9825	0.106*	
H28H	0.4111	1.22	0.9808	0.106*	
H28I	0.5029	1.2288	0.9561	0.106*	
C29B	0.5307 (5)	1.1919 (5)	1.03137 (13)	0.0689 (15)	
H29G	0.5812	1.1556	1.0443	0.103*	
H29H	0.4589	1.1671	1.0354	0.103*	
H29I	0.5343	1.2652	1.0356	0.103*	
C30B	0.5157 (4)	0.7846 (4)	1.01660 (9)	0.0479 (10)	
H30G	0.5132	0.8419	1.0307	0.072*	
H30H	0.5791	0.7436	1.0205	0.072*	
H30I	0.4524	0.7422	1.0192	0.072*	
O1B	0.3378 (3)	0.6324 (3)	0.90038 (8)	0.0638 (10)	
O2B	0.4888 (2)	0.5657 (3)	0.91689 (6)	0.0472 (7)	
O3B	0.4382 (2)	0.6494 (3)	0.97506 (6)	0.0473 (7)	
O4B	0.4197 (3)	0.9291 (3)	0.94662 (6)	0.0457 (7)	
H4OB	0.356 (6)	0.926 (5)	0.9378 (14)	0.077 (19)*	
O5B	0.6974 (4)	1.3024 (3)	0.99746 (13)	0.0910 (15)	
O6B	0.8156 (4)	0.4137 (4)	0.91363 (13)	0.0871 (14)	

### Atomic displacement parameters (Å^2^)

**Table:**

	*U*^11^	*U*^22^	*U*^33^	*U*^12^	*U*^13^	*U*^23^
C1	0.074 (4)	0.042 (3)	0.088 (4)	−0.019 (3)	0.016 (3)	−0.017 (3)
C2	0.079 (4)	0.042 (3)	0.110 (5)	−0.013 (3)	0.013 (4)	−0.008 (3)
C3	0.060 (3)	0.050 (3)	0.065 (3)	−0.008 (2)	0.000 (2)	0.010 (2)
C4	0.041 (2)	0.060 (3)	0.050 (2)	0.000 (2)	−0.0023 (18)	0.004 (2)
C5	0.043 (2)	0.042 (2)	0.0444 (19)	0.0048 (19)	−0.0045 (17)	0.0015 (17)
C6	0.046 (2)	0.039 (2)	0.050 (2)	0.0038 (19)	−0.0041 (18)	−0.0074 (18)
C7	0.047 (2)	0.036 (2)	0.050 (2)	−0.0022 (19)	−0.0042 (18)	−0.0123 (18)
C8	0.053 (2)	0.0269 (18)	0.0400 (18)	−0.0009 (18)	−0.0020 (17)	−0.0012 (15)
C9	0.052 (2)	0.0325 (19)	0.0377 (17)	−0.0053 (19)	−0.0003 (17)	−0.0038 (16)
C10	0.047 (2)	0.037 (2)	0.050 (2)	−0.0079 (19)	−0.0040 (19)	−0.0059 (18)
C11	0.078 (3)	0.048 (3)	0.045 (2)	−0.010 (3)	0.005 (2)	−0.017 (2)
C12	0.068 (3)	0.044 (2)	0.0367 (18)	0.001 (2)	0.0045 (19)	−0.0073 (18)
C13	0.050 (2)	0.035 (2)	0.0405 (18)	0.0029 (18)	0.0060 (17)	−0.0026 (16)
C14	0.055 (2)	0.0248 (18)	0.0446 (19)	−0.0062 (18)	0.0041 (18)	0.0028 (16)
C15	0.056 (3)	0.038 (2)	0.069 (3)	−0.008 (2)	0.011 (2)	−0.011 (2)
C16	0.057 (3)	0.044 (3)	0.072 (3)	−0.012 (2)	0.007 (2)	−0.019 (2)
C17	0.058 (3)	0.052 (3)	0.047 (2)	−0.007 (2)	0.010 (2)	−0.003 (2)
C18	0.060 (3)	0.043 (2)	0.050 (2)	0.004 (2)	0.006 (2)	0.0024 (19)
C19	0.058 (3)	0.077 (4)	0.056 (3)	−0.004 (3)	−0.017 (2)	−0.006 (3)
C20	0.053 (3)	0.068 (3)	0.065 (3)	−0.008 (3)	0.015 (2)	−0.021 (3)
C21	0.072 (4)	0.113 (5)	0.062 (3)	0.006 (4)	0.021 (3)	−0.015 (3)
C22	0.051 (3)	0.088 (4)	0.075 (3)	0.002 (3)	0.002 (3)	−0.031 (3)
C23	0.060 (4)	0.079 (4)	0.128 (6)	0.006 (3)	0.011 (4)	−0.041 (4)
C28	0.053 (3)	0.096 (4)	0.058 (3)	0.002 (3)	0.005 (2)	0.004 (3)
C29	0.051 (3)	0.060 (3)	0.076 (3)	0.006 (2)	−0.006 (2)	0.002 (3)
C30	0.073 (3)	0.045 (3)	0.056 (2)	−0.001 (2)	−0.008 (2)	0.017 (2)
O1	0.069 (2)	0.071 (3)	0.097 (3)	−0.009 (2)	−0.004 (2)	−0.045 (2)
O2	0.059 (2)	0.057 (2)	0.072 (2)	−0.0079 (18)	0.0111 (17)	−0.0232 (18)
O3	0.072 (2)	0.0352 (16)	0.085 (2)	−0.0049 (17)	0.014 (2)	0.0123 (17)
O4	0.0522 (18)	0.064 (2)	0.0378 (14)	0.0053 (17)	−0.0031 (14)	−0.0063 (14)
O5	0.054 (2)	0.076 (3)	0.111 (3)	−0.017 (2)	0.005 (2)	0.015 (2)
O6	0.076 (3)	0.116 (4)	0.105 (3)	0.001 (3)	0.030 (3)	−0.053 (3)
C1A	0.061 (3)	0.071 (3)	0.060 (3)	0.027 (3)	−0.024 (2)	−0.035 (3)
C2A	0.061 (3)	0.093 (4)	0.051 (2)	0.018 (3)	−0.019 (2)	−0.024 (3)
C3A	0.038 (2)	0.058 (3)	0.055 (2)	0.004 (2)	−0.0024 (19)	0.003 (2)
C4A	0.044 (2)	0.055 (3)	0.046 (2)	0.008 (2)	0.0047 (18)	−0.0039 (19)
C5A	0.047 (2)	0.042 (2)	0.0365 (17)	0.0058 (19)	0.0088 (16)	−0.0001 (17)
C6A	0.064 (3)	0.051 (3)	0.0400 (19)	−0.002 (2)	0.010 (2)	−0.0117 (19)
C7A	0.059 (3)	0.041 (2)	0.0299 (16)	−0.010 (2)	0.0030 (17)	−0.0076 (16)
C8A	0.051 (2)	0.036 (2)	0.0345 (17)	−0.0064 (19)	−0.0046 (16)	0.0021 (16)
C9A	0.046 (2)	0.035 (2)	0.0393 (17)	−0.0017 (18)	−0.0075 (16)	−0.0063 (16)
C10A	0.044 (2)	0.039 (2)	0.048 (2)	0.0009 (19)	−0.0101 (18)	−0.0073 (18)
C11A	0.049 (3)	0.036 (2)	0.080 (3)	0.002 (2)	−0.021 (2)	−0.016 (2)
C12A	0.046 (2)	0.034 (2)	0.081 (3)	−0.0051 (19)	−0.022 (2)	0.001 (2)
C13A	0.050 (2)	0.035 (2)	0.046 (2)	−0.0054 (19)	−0.0137 (18)	0.0014 (17)
C14A	0.054 (2)	0.042 (2)	0.0339 (16)	−0.011 (2)	−0.0125 (17)	0.0047 (17)
C15A	0.064 (3)	0.059 (3)	0.0413 (19)	−0.008 (3)	−0.013 (2)	−0.009 (2)
C16A	0.062 (3)	0.058 (3)	0.050 (2)	−0.015 (3)	−0.016 (2)	−0.005 (2)
C17A	0.051 (3)	0.057 (3)	0.066 (3)	−0.011 (2)	−0.024 (2)	0.009 (2)
C18A	0.052 (3)	0.052 (3)	0.045 (2)	0.002 (2)	−0.0010 (18)	0.0026 (19)
C19A	0.043 (3)	0.054 (3)	0.105 (4)	−0.007 (2)	−0.004 (3)	0.014 (3)
C20A	0.048 (3)	0.052 (3)	0.092 (4)	0.002 (2)	−0.023 (3)	0.004 (3)
C21A	0.087 (5)	0.052 (3)	0.123 (5)	0.009 (3)	−0.018 (4)	0.003 (4)
C23A	0.052 (3)	0.052 (3)	0.148 (6)	0.011 (3)	−0.009 (4)	−0.025 (4)
C22A	0.065 (4)	0.071 (4)	0.158 (7)	0.001 (4)	0.002 (4)	−0.023 (5)
C28A	0.062 (3)	0.102 (5)	0.073 (3)	0.018 (3)	0.029 (3)	−0.001 (3)
C29A	0.085 (4)	0.057 (3)	0.058 (3)	0.022 (3)	−0.007 (3)	0.001 (2)
C30A	0.069 (3)	0.064 (3)	0.055 (2)	−0.015 (3)	−0.004 (2)	0.022 (2)
O1A	0.081 (3)	0.069 (2)	0.093 (3)	−0.033 (2)	−0.008 (2)	−0.018 (2)
O2A	0.054 (2)	0.061 (2)	0.077 (2)	−0.0121 (18)	−0.0223 (17)	−0.0044 (19)
O3A	0.069 (2)	0.081 (2)	0.0379 (14)	−0.018 (2)	−0.0184 (15)	0.0142 (16)
O4A	0.070 (2)	0.0401 (17)	0.0472 (15)	−0.0099 (16)	0.0113 (15)	−0.0050 (13)
O5A	0.0462 (19)	0.086 (3)	0.085 (2)	0.016 (2)	−0.0177 (17)	−0.009 (2)
O6A	0.063 (3)	0.117 (5)	0.181 (6)	0.025 (3)	−0.001 (3)	−0.018 (4)
C1B	0.035 (2)	0.073 (4)	0.105 (4)	−0.011 (2)	0.007 (3)	−0.051 (3)
C2B	0.050 (3)	0.063 (3)	0.100 (4)	−0.022 (3)	0.020 (3)	−0.031 (3)
C3B	0.062 (3)	0.048 (3)	0.068 (3)	−0.012 (2)	−0.003 (2)	−0.014 (2)
C4B	0.053 (3)	0.044 (2)	0.058 (2)	0.000 (2)	0.003 (2)	−0.015 (2)
C5B	0.0341 (19)	0.044 (2)	0.0422 (18)	0.0002 (18)	0.0031 (15)	−0.0108 (17)
C6B	0.035 (2)	0.051 (2)	0.046 (2)	0.0056 (19)	0.0036 (17)	−0.0070 (18)
C7B	0.0296 (18)	0.046 (2)	0.0343 (16)	0.0013 (17)	0.0038 (15)	0.0006 (16)
C8B	0.0290 (17)	0.046 (2)	0.0292 (15)	−0.0020 (16)	−0.0021 (14)	−0.0074 (15)
C9B	0.0282 (17)	0.045 (2)	0.0444 (18)	0.0021 (17)	−0.0055 (15)	−0.0149 (18)
C10B	0.033 (2)	0.050 (3)	0.057 (2)	0.0032 (19)	−0.0057 (17)	−0.023 (2)
C11B	0.0281 (19)	0.056 (3)	0.075 (3)	0.010 (2)	−0.011 (2)	−0.024 (2)
C12B	0.039 (2)	0.051 (3)	0.054 (2)	0.007 (2)	−0.0103 (18)	−0.013 (2)
C13B	0.0284 (17)	0.038 (2)	0.0386 (17)	−0.0045 (16)	0.0020 (14)	−0.0044 (16)
C14B	0.0284 (17)	0.038 (2)	0.0334 (16)	−0.0021 (16)	0.0004 (14)	0.0020 (15)
C15B	0.0327 (19)	0.051 (2)	0.0394 (18)	−0.0051 (18)	−0.0015 (15)	−0.0071 (17)
C16B	0.037 (2)	0.053 (3)	0.046 (2)	−0.008 (2)	0.0029 (17)	−0.0072 (19)
C17B	0.0346 (19)	0.039 (2)	0.0421 (18)	−0.0014 (18)	−0.0008 (15)	−0.0022 (16)
C18B	0.049 (2)	0.045 (2)	0.0429 (19)	−0.005 (2)	0.0096 (18)	−0.0012 (18)
C19B	0.057 (3)	0.077 (4)	0.075 (3)	0.016 (3)	−0.030 (3)	−0.032 (3)
C20B	0.043 (2)	0.039 (2)	0.057 (2)	−0.0009 (19)	0.0005 (19)	−0.0065 (19)
C21B	0.065 (3)	0.054 (3)	0.077 (3)	0.022 (3)	−0.009 (3)	−0.017 (3)
C22B	0.065 (3)	0.049 (3)	0.074 (3)	0.009 (3)	0.019 (3)	−0.007 (2)
C23B	0.063 (4)	0.057 (3)	0.109 (5)	−0.002 (3)	0.029 (3)	−0.022 (3)
C28B	0.073 (4)	0.051 (3)	0.089 (4)	0.013 (3)	−0.005 (3)	−0.002 (3)
C29B	0.079 (4)	0.057 (3)	0.071 (3)	−0.004 (3)	0.017 (3)	−0.028 (3)
C30B	0.054 (3)	0.057 (3)	0.0322 (17)	0.004 (2)	−0.0059 (18)	−0.0030 (17)
O1B	0.0500 (19)	0.083 (3)	0.0583 (18)	0.0103 (19)	−0.0159 (16)	−0.0279 (18)
O2B	0.0402 (15)	0.0530 (18)	0.0485 (14)	−0.0010 (14)	−0.0024 (12)	−0.0141 (13)
O3B	0.0439 (16)	0.0572 (18)	0.0407 (13)	−0.0134 (15)	0.0060 (12)	−0.0001 (13)
O4B	0.0355 (15)	0.0604 (19)	0.0412 (13)	0.0014 (15)	−0.0048 (12)	0.0041 (13)
O5B	0.076 (3)	0.055 (2)	0.141 (4)	−0.016 (2)	0.018 (3)	−0.026 (3)
O6B	0.063 (2)	0.066 (3)	0.133 (4)	0.020 (2)	0.000 (3)	−0.032 (3)

### Geometric parameters (Å, °)

**Table:**

C1—C2	1.312 (9)	C14A—O3A	1.460 (4)
C1—C10	1.505 (7)	C14A—C15A	1.486 (6)
C1—H1	0.93	C15A—O3A	1.440 (6)
C2—C3	1.462 (9)	C15A—C16A	1.482 (8)
C2—H2	0.93	C15A—H15A	0.98
C3—O5	1.215 (7)	C16A—O1A	1.213 (6)
C3—C4	1.503 (7)	C16A—O2A	1.330 (7)
C4—C29	1.530 (7)	C17A—O2A	1.475 (6)
C4—C28	1.555 (7)	C17A—C20A	1.482 (8)
C4—C5	1.570 (6)	C17A—H17A	0.98
C5—C6	1.520 (6)	C18A—H18D	0.96
C5—C10	1.552 (6)	C18A—H18E	0.96
C5—H5	0.98	C18A—H18F	0.96
C6—C7	1.532 (7)	C19A—H19D	0.96
C6—H6A	0.97	C19A—H19E	0.96
C6—H6B	0.97	C19A—H19F	0.96
C7—O4	1.431 (5)	C20A—C22A	1.375 (10)
C7—C8	1.534 (6)	C20A—C21A	1.404 (9)
C7—H7	0.98	C21A—C23A	1.316 (11)
C8—C30	1.545 (6)	C21A—H21A	0.93
C8—C9	1.559 (6)	C23A—O6A	1.345 (9)
C8—C14	1.562 (7)	C23A—H23A	0.93
C9—C11	1.533 (6)	C22A—O6A	1.350 (9)
C9—C10	1.577 (7)	C22A—H22A	0.93
C9—H9	0.98	C28A—H28D	0.96
C10—C19	1.544 (7)	C28A—H28E	0.96
C11—C12	1.532 (7)	C28A—H28F	0.96
C11—H11A	0.97	C29A—H29D	0.96
C11—H11B	0.97	C29A—H29E	0.96
C12—C13	1.533 (6)	C29A—H29F	0.96
C12—H12A	0.97	C30A—H30D	0.96
C12—H12B	0.97	C30A—H30E	0.96
C13—C14	1.537 (6)	C30A—H30F	0.96
C13—C17	1.538 (6)	O4A—H4OA	1.01 (6)
C13—C18	1.539 (6)	C1B—C2B	1.328 (9)
C14—O3	1.441 (5)	C1B—C10B	1.510 (7)
C14—C15	1.474 (7)	C1B—H1B	0.93
C15—O3	1.424 (7)	C2B—C3B	1.471 (8)
C15—C16	1.497 (8)	C2B—H2B	0.93
C15—H15	0.98	C3B—O5B	1.213 (6)
C16—O1	1.204 (6)	C3B—C4B	1.509 (8)
C16—O2	1.323 (6)	C4B—C28B	1.534 (8)
C17—O2	1.471 (6)	C4B—C29B	1.537 (7)
C17—C20	1.493 (7)	C4B—C5B	1.549 (6)
C17—H17	0.98	C5B—C6B	1.513 (6)
C18—H18A	0.96	C5B—C10B	1.558 (6)
C18—H18B	0.96	C5B—H5B	0.98
C18—H18C	0.96	C6B—C7B	1.549 (6)
C19—H19A	0.96	C6B—H6B1	0.97
C19—H19B	0.96	C6B—H6B2	0.97
C19—H19C	0.96	C7B—O4B	1.431 (4)
C20—C21	1.365 (7)	C7B—C8B	1.532 (6)
C20—C22	1.412 (9)	C7B—H7B	0.98
C21—O6	1.363 (9)	C8B—C30B	1.545 (5)
C21—H21	0.93	C8B—C9B	1.549 (6)
C22—C23	1.349 (9)	C8B—C14B	1.573 (5)
C22—H22	0.93	C9B—C11B	1.543 (6)
C23—O6	1.359 (10)	C9B—C10B	1.564 (6)
C23—H23	0.93	C9B—H9B	0.98
C28—H28A	0.96	C10B—C19B	1.545 (7)
C28—H28B	0.96	C11B—C12B	1.536 (7)
C28—H28C	0.96	C11B—H11E	0.97
C29—H29A	0.96	C11B—H11F	0.97
C29—H29B	0.96	C12B—C13B	1.533 (6)
C29—H29C	0.96	C12B—H12E	0.97
C30—H30A	0.96	C12B—H12F	0.97
C30—H30B	0.96	C13B—C14B	1.535 (5)
C30—H30C	0.96	C13B—C18B	1.542 (6)
O4—H4O	0.81 (5)	C13B—C17B	1.553 (6)
C1A—C2A	1.333 (7)	C14B—O3B	1.453 (5)
C1A—C10A	1.506 (6)	C14B—C15B	1.458 (5)
C1A—H1A	0.93	C15B—O3B	1.431 (5)
C2A—C3A	1.449 (7)	C15B—C16B	1.491 (6)
C2A—H2A	0.93	C15B—H15B	0.98
C3A—O5A	1.227 (6)	C16B—O1B	1.208 (5)
C3A—C4A	1.533 (6)	C16B—O2B	1.324 (6)
C4A—C29A	1.539 (8)	C17B—O2B	1.465 (5)
C4A—C28A	1.544 (7)	C17B—C20B	1.499 (6)
C4A—C5A	1.554 (6)	C17B—H17B	0.98
C5A—C6A	1.532 (6)	C18B—H18G	0.96
C5A—C10A	1.543 (6)	C18B—H18H	0.96
C5A—H5A	0.98	C18B—H18I	0.96
C6A—C7A	1.520 (7)	C19B—H19G	0.96
C6A—H6A1	0.97	C19B—H19H	0.96
C6A—H6A2	0.97	C19B—H19I	0.96
C7A—O4A	1.416 (5)	C20B—C21B	1.337 (7)
C7A—C8A	1.528 (6)	C20B—C22B	1.432 (7)
C7A—H7A	0.98	C21B—O6B	1.377 (7)
C8A—C30A	1.545 (6)	C21B—H21B	0.93
C8A—C14A	1.557 (7)	C22B—C23B	1.339 (8)
C8A—C9A	1.563 (5)	C22B—H22B	0.93
C9A—C11A	1.539 (6)	C23B—O6B	1.354 (9)
C9A—C10A	1.573 (6)	C23B—H23B	0.93
C9A—H9A	1.1028	C28B—H28G	0.96
C10A—C19A	1.536 (7)	C28B—H28H	0.96
C11A—C12A	1.554 (6)	C28B—H28I	0.96
C11A—H11C	0.97	C29B—H29G	0.96
C11A—H11D	0.97	C29B—H29H	0.96
C12A—C13A	1.527 (6)	C29B—H29I	0.96
C12A—H12C	0.97	C30B—H30G	0.96
C12A—H12D	0.97	C30B—H30H	0.96
C13A—C14A	1.516 (6)	C30B—H30I	0.96
C13A—C17A	1.552 (7)	O4B—H4OB	0.88 (7)
C13A—C18A	1.556 (6)		
			
C2—C1—C10	120.9 (5)	C17A—C13A—C18A	107.4 (4)
C2—C1—H1	119.5	O3A—C14A—C15A	58.5 (3)
C10—C1—H1	119.5	O3A—C14A—C13A	112.7 (4)
C1—C2—C3	123.3 (5)	C15A—C14A—C13A	114.2 (4)
C1—C2—H2	118.4	O3A—C14A—C8A	113.7 (4)
C3—C2—H2	118.4	C15A—C14A—C8A	122.1 (4)
O5—C3—C2	120.4 (5)	C13A—C14A—C8A	119.7 (3)
O5—C3—C4	121.2 (5)	O3A—C15A—C16A	113.1 (5)
C2—C3—C4	118.3 (5)	O3A—C15A—C14A	59.9 (3)
C3—C4—C29	108.7 (4)	C16A—C15A—C14A	118.4 (4)
C3—C4—C28	105.7 (4)	O3A—C15A—H15A	117.5
C29—C4—C28	108.7 (4)	C16A—C15A—H15A	117.5
C3—C4—C5	111.1 (4)	C14A—C15A—H15A	117.5
C29—C4—C5	114.4 (4)	O1A—C16A—O2A	119.0 (5)
C28—C4—C5	107.9 (4)	O1A—C16A—C15A	122.1 (6)
C6—C5—C10	111.7 (4)	O2A—C16A—C15A	118.9 (5)
C6—C5—C4	113.7 (4)	O2A—C17A—C20A	104.6 (4)
C10—C5—C4	114.5 (4)	O2A—C17A—C13A	110.4 (4)
C6—C5—H5	105.3	C20A—C17A—C13A	116.1 (4)
C10—C5—H5	105.3	O2A—C17A—H17A	108.5
C4—C5—H5	105.3	C20A—C17A—H17A	108.5
C5—C6—C7	111.5 (4)	C13A—C17A—H17A	108.5
C5—C6—H6A	109.3	C13A—C18A—H18D	109.5
C7—C6—H6A	109.3	C13A—C18A—H18E	109.5
C5—C6—H6B	109.3	H18D—C18A—H18E	109.5
C7—C6—H6B	109.3	C13A—C18A—H18F	109.5
H6A—C6—H6B	108	H18D—C18A—H18F	109.5
O4—C7—C6	110.7 (4)	H18E—C18A—H18F	109.5
O4—C7—C8	107.2 (3)	C10A—C19A—H19D	109.5
C6—C7—C8	111.6 (3)	C10A—C19A—H19E	109.5
O4—C7—H7	109.1	H19D—C19A—H19E	109.5
C6—C7—H7	109.1	C10A—C19A—H19F	109.5
C8—C7—H7	109.1	H19D—C19A—H19F	109.5
C7—C8—C30	109.5 (4)	H19E—C19A—H19F	109.5
C7—C8—C9	109.0 (3)	C22A—C20A—C21A	103.6 (7)
C30—C8—C9	112.4 (4)	C22A—C20A—C17A	127.6 (6)
C7—C8—C14	110.5 (3)	C21A—C20A—C17A	128.8 (6)
C30—C8—C14	106.3 (4)	C23A—C21A—C20A	110.0 (7)
C9—C8—C14	109.1 (3)	C23A—C21A—H21A	125
C11—C9—C8	111.2 (3)	C20A—C21A—H21A	125
C11—C9—C10	113.9 (4)	C21A—C23A—O6A	108.6 (6)
C8—C9—C10	115.4 (4)	C21A—C23A—H23A	125.7
C11—C9—H9	105	O6A—C23A—H23A	125.7
C8—C9—H9	105	O6A—C22A—C20A	109.7 (7)
C10—C9—H9	105	O6A—C22A—H22A	125.2
C1—C10—C19	105.1 (4)	C20A—C22A—H22A	125.2
C1—C10—C5	106.0 (4)	C4A—C28A—H28D	109.5
C19—C10—C5	114.9 (4)	C4A—C28A—H28E	109.5
C1—C10—C9	109.1 (4)	H28D—C28A—H28E	109.5
C19—C10—C9	114.7 (4)	C4A—C28A—H28F	109.5
C5—C10—C9	106.6 (3)	H28D—C28A—H28F	109.5
C12—C11—C9	115.3 (4)	H28E—C28A—H28F	109.5
C12—C11—H11A	108.5	C4A—C29A—H29D	109.5
C9—C11—H11A	108.5	C4A—C29A—H29E	109.5
C12—C11—H11B	108.5	H29D—C29A—H29E	109.5
C9—C11—H11B	108.5	C4A—C29A—H29F	109.5
H11A—C11—H11B	107.5	H29D—C29A—H29F	109.5
C11—C12—C13	113.1 (3)	H29E—C29A—H29F	109.5
C11—C12—H12A	109	C8A—C30A—H30D	109.5
C13—C12—H12A	109	C8A—C30A—H30E	109.5
C11—C12—H12B	109	H30D—C30A—H30E	109.5
C13—C12—H12B	109	C8A—C30A—H30F	109.5
H12A—C12—H12B	107.8	H30D—C30A—H30F	109.5
C12—C13—C14	105.4 (4)	H30E—C30A—H30F	109.5
C12—C13—C17	109.3 (3)	C16A—O2A—C17A	120.7 (4)
C14—C13—C17	107.3 (4)	C15A—O3A—C14A	61.6 (3)
C12—C13—C18	113.7 (4)	C7A—O4A—H4OA	101 (3)
C14—C13—C18	111.6 (3)	C23A—O6A—C22A	108.0 (6)
C17—C13—C18	109.3 (4)	C2B—C1B—C10B	122.9 (5)
O3—C14—C15	58.5 (3)	C2B—C1B—H1B	118.6
O3—C14—C13	112.7 (3)	C10B—C1B—H1B	118.6
C15—C14—C13	114.3 (4)	C1B—C2B—C3B	122.3 (5)
O3—C14—C8	113.4 (4)	C1B—C2B—H2B	118.9
C15—C14—C8	123.0 (4)	C3B—C2B—H2B	118.9
C13—C14—C8	119.2 (3)	O5B—C3B—C2B	118.8 (5)
O3—C15—C14	59.6 (3)	O5B—C3B—C4B	122.3 (5)
O3—C15—C16	113.1 (4)	C2B—C3B—C4B	118.9 (4)
C14—C15—C16	119.0 (4)	C3B—C4B—C28B	105.1 (5)
O3—C15—H15	117.4	C3B—C4B—C29B	108.4 (4)
C14—C15—H15	117.4	C28B—C4B—C29B	108.8 (5)
C16—C15—H15	117.4	C3B—C4B—C5B	111.5 (4)
O1—C16—O2	119.8 (5)	C28B—C4B—C5B	108.2 (4)
O1—C16—C15	121.8 (5)	C29B—C4B—C5B	114.4 (4)
O2—C16—C15	118.4 (5)	C6B—C5B—C4B	114.5 (4)
O2—C17—C20	104.6 (4)	C6B—C5B—C10B	111.7 (4)
O2—C17—C13	110.9 (3)	C4B—C5B—C10B	115.8 (4)
C20—C17—C13	115.5 (4)	C6B—C5B—H5B	104.4
O2—C17—H17	108.5	C4B—C5B—H5B	104.4
C20—C17—H17	108.5	C10B—C5B—H5B	104.4
C13—C17—H17	108.5	C5B—C6B—C7B	110.2 (3)
C13—C18—H18A	109.5	C5B—C6B—H6B1	109.6
C13—C18—H18B	109.5	C7B—C6B—H6B1	109.6
H18A—C18—H18B	109.5	C5B—C6B—H6B2	109.6
C13—C18—H18C	109.5	C7B—C6B—H6B2	109.6
H18A—C18—H18C	109.5	H6B1—C6B—H6B2	108.1
H18B—C18—H18C	109.5	O4B—C7B—C8B	108.9 (3)
C10—C19—H19A	109.5	O4B—C7B—C6B	108.0 (3)
C10—C19—H19B	109.5	C8B—C7B—C6B	112.3 (3)
H19A—C19—H19B	109.5	O4B—C7B—H7B	109.2
C10—C19—H19C	109.5	C8B—C7B—H7B	109.2
H19A—C19—H19C	109.5	C6B—C7B—H7B	109.2
H19B—C19—H19C	109.5	C7B—C8B—C30B	109.4 (3)
C21—C20—C22	105.4 (5)	C7B—C8B—C9B	108.8 (3)
C21—C20—C17	125.4 (6)	C30B—C8B—C9B	112.7 (3)
C22—C20—C17	129.2 (5)	C7B—C8B—C14B	110.3 (3)
O6—C21—C20	110.6 (6)	C30B—C8B—C14B	106.5 (3)
O6—C21—H21	124.7	C9B—C8B—C14B	109.1 (3)
C20—C21—H21	124.7	C11B—C9B—C8B	111.9 (4)
C23—C22—C20	107.4 (6)	C11B—C9B—C10B	113.4 (3)
C23—C22—H22	126.3	C8B—C9B—C10B	115.0 (3)
C20—C22—H22	126.3	C11B—C9B—H9B	105.1
C22—C23—O6	110.2 (6)	C8B—C9B—H9B	105.1
C22—C23—H23	124.9	C10B—C9B—H9B	105.1
O6—C23—H23	124.9	C1B—C10B—C19B	105.2 (4)
C4—C28—H28A	109.5	C1B—C10B—C5B	106.1 (4)
C4—C28—H28B	109.5	C19B—C10B—C5B	113.3 (3)
H28A—C28—H28B	109.5	C1B—C10B—C9B	108.3 (4)
C4—C28—H28C	109.5	C19B—C10B—C9B	116.2 (4)
H28A—C28—H28C	109.5	C5B—C10B—C9B	107.1 (3)
H28B—C28—H28C	109.5	C12B—C11B—C9B	115.6 (3)
C4—C29—H29A	109.5	C12B—C11B—H11E	108.4
C4—C29—H29B	109.5	C9B—C11B—H11E	108.4
H29A—C29—H29B	109.5	C12B—C11B—H11F	108.4
C4—C29—H29C	109.5	C9B—C11B—H11F	108.4
H29A—C29—H29C	109.5	H11E—C11B—H11F	107.4
H29B—C29—H29C	109.5	C13B—C12B—C11B	111.5 (4)
C8—C30—H30A	109.5	C13B—C12B—H12E	109.3
C8—C30—H30B	109.5	C11B—C12B—H12E	109.3
H30A—C30—H30B	109.5	C13B—C12B—H12F	109.3
C8—C30—H30C	109.5	C11B—C12B—H12F	109.3
H30A—C30—H30C	109.5	H12E—C12B—H12F	108
H30B—C30—H30C	109.5	C12B—C13B—C14B	107.1 (3)
C16—O2—C17	120.7 (4)	C12B—C13B—C18B	112.6 (4)
C15—O3—C14	61.9 (3)	C14B—C13B—C18B	111.3 (3)
C7—O4—H4O	103 (3)	C12B—C13B—C17B	109.2 (3)
C23—O6—C21	106.3 (5)	C14B—C13B—C17B	106.8 (3)
C2A—C1A—C10A	122.7 (4)	C18B—C13B—C17B	109.8 (3)
C2A—C1A—H1A	118.7	O3B—C14B—C15B	58.9 (3)
C10A—C1A—H1A	118.7	O3B—C14B—C13B	114.1 (3)
C1A—C2A—C3A	122.3 (4)	C15B—C14B—C13B	114.2 (3)
C1A—C2A—H2A	118.8	O3B—C14B—C8B	112.5 (3)
C3A—C2A—H2A	118.8	C15B—C14B—C8B	123.7 (3)
O5A—C3A—C2A	119.5 (4)	C13B—C14B—C8B	118.4 (3)
O5A—C3A—C4A	120.2 (4)	O3B—C15B—C14B	60.4 (2)
C2A—C3A—C4A	120.2 (4)	O3B—C15B—C16B	113.3 (4)
C3A—C4A—C29A	105.1 (4)	C14B—C15B—C16B	118.2 (4)
C3A—C4A—C28A	108.8 (4)	O3B—C15B—H15B	117.5
C29A—C4A—C28A	109.7 (5)	C14B—C15B—H15B	117.5
C3A—C4A—C5A	109.6 (4)	C16B—C15B—H15B	117.4
C29A—C4A—C5A	108.2 (4)	O1B—C16B—O2B	118.5 (4)
C28A—C4A—C5A	114.9 (4)	O1B—C16B—C15B	122.8 (4)
C6A—C5A—C10A	110.7 (4)	O2B—C16B—C15B	118.7 (4)
C6A—C5A—C4A	114.8 (4)	O2B—C17B—C20B	105.9 (3)
C10A—C5A—C4A	116.7 (4)	O2B—C17B—C13B	109.5 (3)
C6A—C5A—H5A	104.3	C20B—C17B—C13B	115.7 (3)
C10A—C5A—H5A	104.3	O2B—C17B—H17B	108.5
C4A—C5A—H5A	104.3	C20B—C17B—H17B	108.5
C7A—C6A—C5A	110.9 (3)	C13B—C17B—H17B	108.5
C7A—C6A—H6A1	109.5	C13B—C18B—H18G	109.5
C5A—C6A—H6A1	109.5	C13B—C18B—H18H	109.5
C7A—C6A—H6A2	109.5	H18G—C18B—H18H	109.5
C5A—C6A—H6A2	109.5	C13B—C18B—H18I	109.5
H6A1—C6A—H6A2	108.1	H18G—C18B—H18I	109.5
O4A—C7A—C6A	109.7 (4)	H18H—C18B—H18I	109.5
O4A—C7A—C8A	108.6 (3)	C10B—C19B—H19G	109.5
C6A—C7A—C8A	112.6 (4)	C10B—C19B—H19H	109.5
O4A—C7A—H7A	108.6	H19G—C19B—H19H	109.5
C6A—C7A—H7A	108.6	C10B—C19B—H19I	109.5
C8A—C7A—H7A	108.6	H19G—C19B—H19I	109.5
C7A—C8A—C30A	109.8 (4)	H19H—C19B—H19I	109.5
C7A—C8A—C14A	110.7 (4)	C21B—C20B—C22B	106.7 (5)
C30A—C8A—C14A	107.0 (4)	C21B—C20B—C17B	124.8 (4)
C7A—C8A—C9A	109.1 (3)	C22B—C20B—C17B	128.5 (4)
C30A—C8A—C9A	112.4 (4)	C20B—C21B—O6B	110.0 (5)
C14A—C8A—C9A	107.9 (3)	C20B—C21B—H21B	125
C11A—C9A—C8A	111.3 (4)	O6B—C21B—H21B	125
C11A—C9A—C10A	114.1 (4)	C23B—C22B—C20B	105.9 (6)
C8A—C9A—C10A	115.5 (3)	C23B—C22B—H22B	127.1
C11A—C9A—H9A	104.9	C20B—C22B—H22B	127.1
C8A—C9A—H9A	104.9	C22B—C23B—O6B	111.2 (5)
C10A—C9A—H9A	104.9	C22B—C23B—H23B	124.4
C1A—C10A—C19A	105.7 (4)	O6B—C23B—H23B	124.4
C1A—C10A—C5A	106.9 (4)	C4B—C28B—H28G	109.5
C19A—C10A—C5A	113.9 (4)	C4B—C28B—H28H	109.5
C1A—C10A—C9A	107.9 (4)	H28G—C28B—H28H	109.5
C19A—C10A—C9A	114.7 (4)	C4B—C28B—H28I	109.5
C5A—C10A—C9A	107.3 (3)	H28G—C28B—H28I	109.5
C9A—C11A—C12A	114.4 (4)	H28H—C28B—H28I	109.5
C9A—C11A—H11C	108.7	C4B—C29B—H29G	109.5
C12A—C11A—H11C	108.7	C4B—C29B—H29H	109.5
C9A—C11A—H11D	108.7	H29G—C29B—H29H	109.5
C12A—C11A—H11D	108.7	C4B—C29B—H29I	109.5
H11C—C11A—H11D	107.6	H29G—C29B—H29I	109.5
C13A—C12A—C11A	112.7 (4)	H29H—C29B—H29I	109.5
C13A—C12A—H12C	109.1	C8B—C30B—H30G	109.5
C11A—C12A—H12C	109.1	C8B—C30B—H30H	109.5
C13A—C12A—H12D	109.1	H30G—C30B—H30H	109.5
C11A—C12A—H12D	109.1	C8B—C30B—H30I	109.5
H12C—C12A—H12D	107.8	H30G—C30B—H30I	109.5
C14A—C13A—C12A	107.6 (4)	H30H—C30B—H30I	109.5
C14A—C13A—C17A	108.3 (3)	C16B—O2B—C17B	120.7 (3)
C12A—C13A—C17A	109.2 (4)	C15B—O3B—C14B	60.8 (2)
C14A—C13A—C18A	111.3 (4)	C7B—O4B—H4OB	118 (4)
C12A—C13A—C18A	113.0 (4)	C23B—O6B—C21B	106.2 (4)
			
C10—C1—C2—C3	0.7 (10)	C12A—C13A—C14A—O3A	−95.7 (4)
C1—C2—C3—O5	155.8 (7)	C17A—C13A—C14A—O3A	22.2 (5)
C1—C2—C3—C4	−25.4 (9)	C18A—C13A—C14A—O3A	140.1 (4)
O5—C3—C4—C29	−49.6 (6)	C12A—C13A—C14A—C15A	−160.0 (4)
C2—C3—C4—C29	131.7 (5)	C17A—C13A—C14A—C15A	−42.2 (5)
O5—C3—C4—C28	67.0 (6)	C18A—C13A—C14A—C15A	75.7 (5)
C2—C3—C4—C28	−111.8 (6)	C12A—C13A—C14A—C8A	42.0 (5)
O5—C3—C4—C5	−176.2 (5)	C17A—C13A—C14A—C8A	159.9 (4)
C2—C3—C4—C5	5.0 (6)	C18A—C13A—C14A—C8A	−82.2 (5)
C3—C4—C5—C6	165.9 (4)	C7A—C8A—C14A—O3A	−89.1 (4)
C29—C4—C5—C6	42.4 (5)	C30A—C8A—C14A—O3A	30.5 (5)
C28—C4—C5—C6	−78.6 (5)	C9A—C8A—C14A—O3A	151.6 (3)
C3—C4—C5—C10	35.7 (5)	C7A—C8A—C14A—C15A	−22.6 (5)
C29—C4—C5—C10	−87.7 (5)	C30A—C8A—C14A—C15A	97.0 (5)
C28—C4—C5—C10	151.2 (4)	C9A—C8A—C14A—C15A	−141.9 (4)
C10—C5—C6—C7	−60.8 (5)	C7A—C8A—C14A—C13A	133.6 (4)
C4—C5—C6—C7	167.6 (3)	C30A—C8A—C14A—C13A	−106.8 (4)
C5—C6—C7—O4	−60.8 (5)	C9A—C8A—C14A—C13A	14.3 (5)
C5—C6—C7—C8	58.6 (5)	C13A—C14A—C15A—O3A	102.7 (4)
O4—C7—C8—C30	−168.4 (4)	C8A—C14A—C15A—O3A	−100.0 (4)
C6—C7—C8—C30	70.1 (5)	O3A—C14A—C15A—C16A	−101.7 (5)
O4—C7—C8—C9	68.2 (4)	C13A—C14A—C15A—C16A	1.0 (6)
C6—C7—C8—C9	−53.2 (5)	C8A—C14A—C15A—C16A	158.3 (4)
O4—C7—C8—C14	−51.7 (4)	O3A—C15A—C16A—O1A	140.7 (5)
C6—C7—C8—C14	−173.1 (3)	C14A—C15A—C16A—O1A	−152.2 (5)
C7—C8—C9—C11	−174.8 (4)	O3A—C15A—C16A—O2A	−38.9 (5)
C30—C8—C9—C11	63.6 (5)	C14A—C15A—C16A—O2A	28.2 (7)
C14—C8—C9—C11	−54.1 (5)	C14A—C13A—C17A—O2A	58.8 (5)
C7—C8—C9—C10	53.4 (4)	C12A—C13A—C17A—O2A	175.7 (4)
C30—C8—C9—C10	−68.2 (5)	C18A—C13A—C17A—O2A	−61.5 (5)
C14—C8—C9—C10	174.2 (3)	C14A—C13A—C17A—C20A	177.7 (4)
C2—C1—C10—C19	−83.3 (7)	C12A—C13A—C17A—C20A	−65.5 (6)
C2—C1—C10—C5	38.8 (7)	C18A—C13A—C17A—C20A	57.3 (5)
C2—C1—C10—C9	153.3 (6)	O2A—C17A—C20A—C22A	28.2 (8)
C6—C5—C10—C1	172.4 (4)	C13A—C17A—C20A—C22A	−93.8 (7)
C4—C5—C10—C1	−56.5 (5)	O2A—C17A—C20A—C21A	−152.9 (6)
C6—C5—C10—C19	−72.0 (5)	C13A—C17A—C20A—C21A	85.1 (7)
C4—C5—C10—C19	59.1 (5)	C22A—C20A—C21A—C23A	0.0 (8)
C6—C5—C10—C9	56.2 (5)	C17A—C20A—C21A—C23A	−179.2 (6)
C4—C5—C10—C9	−172.7 (4)	C20A—C21A—C23A—O6A	−1.2 (9)
C11—C9—C10—C1	61.3 (5)	C21A—C20A—C22A—O6A	1.3 (8)
C8—C9—C10—C1	−168.3 (4)	C17A—C20A—C22A—O6A	−179.6 (6)
C11—C9—C10—C19	−56.3 (5)	O1A—C16A—O2A—C17A	170.3 (4)
C8—C9—C10—C19	74.2 (5)	C15A—C16A—O2A—C17A	−10.1 (6)
C11—C9—C10—C5	175.4 (4)	C20A—C17A—O2A—C16A	−159.3 (4)
C8—C9—C10—C5	−54.2 (4)	C13A—C17A—O2A—C16A	−33.7 (6)
C8—C9—C11—C12	37.1 (6)	C16A—C15A—O3A—C14A	110.4 (4)
C10—C9—C11—C12	169.6 (4)	C13A—C14A—O3A—C15A	−105.4 (4)
C9—C11—C12—C13	22.8 (6)	C8A—C14A—O3A—C15A	114.3 (5)
C11—C12—C13—C14	−61.3 (5)	C21A—C23A—O6A—C22A	2.0 (9)
C11—C12—C13—C17	−176.3 (4)	C20A—C22A—O6A—C23A	−2.0 (9)
C11—C12—C13—C18	61.3 (5)	C10B—C1B—C2B—C3B	1.8 (10)
C12—C13—C14—O3	−93.9 (4)	C1B—C2B—C3B—O5B	160.3 (7)
C17—C13—C14—O3	22.5 (5)	C1B—C2B—C3B—C4B	−20.5 (9)
C18—C13—C14—O3	142.2 (4)	O5B—C3B—C4B—C28B	61.3 (7)
C12—C13—C14—C15	−158.2 (4)	C2B—C3B—C4B—C28B	−117.9 (6)
C17—C13—C14—C15	−41.9 (5)	O5B—C3B—C4B—C29B	−54.9 (8)
C18—C13—C14—C15	77.9 (5)	C2B—C3B—C4B—C29B	125.9 (6)
C12—C13—C14—C8	42.6 (5)	O5B—C3B—C4B—C5B	178.2 (5)
C17—C13—C14—C8	158.9 (4)	C2B—C3B—C4B—C5B	−1.0 (7)
C18—C13—C14—C8	−81.3 (5)	C3B—C4B—C5B—C6B	171.0 (4)
C7—C8—C14—O3	−90.7 (4)	C28B—C4B—C5B—C6B	−73.9 (5)
C30—C8—C14—O3	28.0 (5)	C29B—C4B—C5B—C6B	47.4 (6)
C9—C8—C14—O3	149.5 (3)	C3B—C4B—C5B—C10B	38.7 (5)
C7—C8—C14—C15	−24.2 (5)	C28B—C4B—C5B—C10B	153.8 (4)
C30—C8—C14—C15	94.6 (5)	C29B—C4B—C5B—C10B	−84.8 (5)
C9—C8—C14—C15	−144.0 (4)	C4B—C5B—C6B—C7B	165.6 (4)
C7—C8—C14—C13	133.1 (4)	C10B—C5B—C6B—C7B	−60.2 (4)
C30—C8—C14—C13	−108.1 (4)	C5B—C6B—C7B—O4B	−61.6 (4)
C9—C8—C14—C13	13.3 (5)	C5B—C6B—C7B—C8B	58.4 (4)
C13—C14—C15—O3	102.7 (4)	O4B—C7B—C8B—C30B	−170.6 (3)
C8—C14—C15—O3	−99.0 (4)	C6B—C7B—C8B—C30B	69.8 (4)
O3—C14—C15—C16	−101.2 (5)	O4B—C7B—C8B—C9B	65.8 (4)
C13—C14—C15—C16	1.4 (6)	C6B—C7B—C8B—C9B	−53.7 (4)
C8—C14—C15—C16	159.8 (4)	O4B—C7B—C8B—C14B	−53.8 (4)
O3—C15—C16—O1	138.1 (5)	C6B—C7B—C8B—C14B	−173.4 (3)
C14—C15—C16—O1	−155.1 (5)	C7B—C8B—C9B—C11B	−174.8 (3)
O3—C15—C16—O2	−41.3 (6)	C30B—C8B—C9B—C11B	63.7 (4)
C14—C15—C16—O2	25.6 (7)	C14B—C8B—C9B—C11B	−54.3 (4)
C12—C13—C17—O2	173.9 (4)	C7B—C8B—C9B—C10B	53.9 (4)
C14—C13—C17—O2	60.2 (5)	C30B—C8B—C9B—C10B	−67.6 (5)
C18—C13—C17—O2	−61.0 (5)	C14B—C8B—C9B—C10B	174.4 (3)
C12—C13—C17—C20	−67.3 (5)	C2B—C1B—C10B—C19B	−86.7 (7)
C14—C13—C17—C20	179.0 (4)	C2B—C1B—C10B—C5B	33.7 (7)
C18—C13—C17—C20	57.8 (5)	C2B—C1B—C10B—C9B	148.5 (6)
O2—C17—C20—C21	−142.9 (6)	C6B—C5B—C10B—C1B	172.7 (4)
C13—C17—C20—C21	94.9 (7)	C4B—C5B—C10B—C1B	−53.8 (5)
O2—C17—C20—C22	36.0 (7)	C6B—C5B—C10B—C19B	−72.3 (5)
C13—C17—C20—C22	−86.2 (7)	C4B—C5B—C10B—C19B	61.2 (5)
C22—C20—C21—O6	1.2 (7)	C6B—C5B—C10B—C9B	57.1 (4)
C17—C20—C21—O6	−179.6 (5)	C4B—C5B—C10B—C9B	−169.3 (4)
C21—C20—C22—C23	−0.3 (7)	C11B—C9B—C10B—C1B	60.4 (5)
C17—C20—C22—C23	−179.4 (6)	C8B—C9B—C10B—C1B	−169.0 (4)
C20—C22—C23—O6	−0.7 (8)	C11B—C9B—C10B—C19B	−57.7 (5)
O1—C16—O2—C17	174.5 (5)	C8B—C9B—C10B—C19B	72.9 (5)
C15—C16—O2—C17	−6.1 (7)	C11B—C9B—C10B—C5B	174.5 (4)
C20—C17—O2—C16	−162.8 (5)	C8B—C9B—C10B—C5B	−54.9 (5)
C13—C17—O2—C16	−37.6 (6)	C8B—C9B—C11B—C12B	33.0 (5)
C16—C15—O3—C14	111.1 (5)	C10B—C9B—C11B—C12B	165.2 (4)
C13—C14—O3—C15	−105.5 (4)	C9B—C11B—C12B—C13B	27.0 (6)
C8—C14—O3—C15	115.5 (4)	C11B—C12B—C13B—C14B	−62.4 (5)
C22—C23—O6—C21	1.5 (8)	C11B—C12B—C13B—C18B	60.2 (5)
C20—C21—O6—C23	−1.7 (8)	C11B—C12B—C13B—C17B	−177.6 (4)
C10A—C1A—C2A—C3A	1.9 (10)	C12B—C13B—C14B—O3B	−96.2 (4)
C1A—C2A—C3A—O5A	160.9 (6)	C18B—C13B—C14B—O3B	140.4 (3)
C1A—C2A—C3A—C4A	−18.5 (9)	C17B—C13B—C14B—O3B	20.6 (4)
O5A—C3A—C4A—C29A	61.6 (6)	C12B—C13B—C14B—C15B	−161.3 (4)
C2A—C3A—C4A—C29A	−118.9 (6)	C18B—C13B—C14B—C15B	75.2 (4)
O5A—C3A—C4A—C28A	−55.8 (7)	C17B—C13B—C14B—C15B	−44.5 (4)
C2A—C3A—C4A—C28A	123.6 (6)	C12B—C13B—C14B—C8B	39.6 (5)
O5A—C3A—C4A—C5A	177.7 (5)	C18B—C13B—C14B—C8B	−83.8 (4)
C2A—C3A—C4A—C5A	−2.8 (6)	C17B—C13B—C14B—C8B	156.4 (3)
C3A—C4A—C5A—C6A	171.5 (4)	C7B—C8B—C14B—O3B	−86.6 (4)
C29A—C4A—C5A—C6A	−74.4 (5)	C30B—C8B—C14B—O3B	32.0 (4)
C28A—C4A—C5A—C6A	48.6 (6)	C9B—C8B—C14B—O3B	153.9 (3)
C3A—C4A—C5A—C10A	39.5 (5)	C7B—C8B—C14B—C15B	−20.0 (5)
C29A—C4A—C5A—C10A	153.6 (4)	C30B—C8B—C14B—C15B	98.7 (4)
C28A—C4A—C5A—C10A	−83.4 (5)	C9B—C8B—C14B—C15B	−139.5 (4)
C10A—C5A—C6A—C7A	−61.9 (5)	C7B—C8B—C14B—C13B	136.9 (3)
C4A—C5A—C6A—C7A	163.3 (4)	C30B—C8B—C14B—C13B	−104.4 (4)
C5A—C6A—C7A—O4A	−61.8 (5)	C9B—C8B—C14B—C13B	17.5 (4)
C5A—C6A—C7A—C8A	59.2 (5)	C13B—C14B—C15B—O3B	104.5 (4)
O4A—C7A—C8A—C30A	−167.0 (4)	C8B—C14B—C15B—O3B	−97.7 (4)
C6A—C7A—C8A—C30A	71.3 (4)	O3B—C14B—C15B—C16B	−102.3 (4)
O4A—C7A—C8A—C14A	−49.1 (4)	C13B—C14B—C15B—C16B	2.2 (5)
C6A—C7A—C8A—C14A	−170.8 (3)	C8B—C14B—C15B—C16B	160.0 (4)
O4A—C7A—C8A—C9A	69.5 (4)	O3B—C15B—C16B—O1B	140.6 (5)
C6A—C7A—C8A—C9A	−52.2 (4)	C14B—C15B—C16B—O1B	−151.7 (5)
C7A—C8A—C9A—C11A	−176.7 (4)	O3B—C15B—C16B—O2B	−39.4 (5)
C30A—C8A—C9A—C11A	61.3 (5)	C14B—C15B—C16B—O2B	28.3 (6)
C14A—C8A—C9A—C11A	−56.3 (5)	C12B—C13B—C17B—O2B	176.7 (3)
C7A—C8A—C9A—C10A	51.2 (5)	C14B—C13B—C17B—O2B	61.3 (4)
C30A—C8A—C9A—C10A	−70.8 (5)	C18B—C13B—C17B—O2B	−59.4 (4)
C14A—C8A—C9A—C10A	171.5 (3)	C12B—C13B—C17B—C20B	−63.7 (4)
C2A—C1A—C10A—C19A	−89.2 (7)	C14B—C13B—C17B—C20B	−179.1 (3)
C2A—C1A—C10A—C5A	32.4 (8)	C18B—C13B—C17B—C20B	60.2 (4)
C2A—C1A—C10A—C9A	147.6 (6)	O2B—C17B—C20B—C21B	−137.4 (5)
C6A—C5A—C10A—C1A	172.7 (4)	C13B—C17B—C20B—C21B	101.0 (6)
C4A—C5A—C10A—C1A	−53.4 (5)	O2B—C17B—C20B—C22B	43.8 (6)
C6A—C5A—C10A—C19A	−71.0 (5)	C13B—C17B—C20B—C22B	−77.7 (6)
C4A—C5A—C10A—C19A	62.9 (5)	C22B—C20B—C21B—O6B	1.3 (6)
C6A—C5A—C10A—C9A	57.1 (4)	C17B—C20B—C21B—O6B	−177.7 (4)
C4A—C5A—C10A—C9A	−169.0 (3)	C21B—C20B—C22B—C23B	−1.0 (6)
C11A—C9A—C10A—C1A	60.6 (5)	C17B—C20B—C22B—C23B	178.0 (5)
C8A—C9A—C10A—C1A	−168.6 (4)	C20B—C22B—C23B—O6B	0.3 (7)
C11A—C9A—C10A—C19A	−56.9 (5)	O1B—C16B—O2B—C17B	170.2 (4)
C8A—C9A—C10A—C19A	73.9 (5)	C15B—C16B—O2B—C17B	−9.8 (6)
C11A—C9A—C10A—C5A	175.5 (4)	C20B—C17B—O2B—C16B	−161.0 (4)
C8A—C9A—C10A—C5A	−53.7 (4)	C13B—C17B—O2B—C16B	−35.5 (5)
C8A—C9A—C11A—C12A	41.0 (6)	C16B—C15B—O3B—C14B	110.2 (4)
C10A—C9A—C11A—C12A	173.8 (4)	C13B—C14B—O3B—C15B	−104.8 (3)
C9A—C11A—C12A—C13A	18.1 (7)	C8B—C14B—O3B—C15B	116.8 (4)
C11A—C12A—C13A—C14A	−58.5 (5)	C22B—C23B—O6B—C21B	0.5 (7)
C11A—C12A—C13A—C17A	−175.9 (4)	C20B—C21B—O6B—C23B	−1.1 (6)
C11A—C12A—C13A—C18A	64.7 (6)		

### Hydrogen-bond geometry (Å, °)

**Table:**

*D*—H···*A*	*D*—H	H···*A*	*D*···*A*	*D*—H···*A*
O4A—H4OA···O1	1.01 (6)	1.83 (6)	2.818 (5)	169 (5)
O4—H4O···O1B	0.81 (5)	2.14 (5)	2.952 (5)	177 (4)
O4B—H4OB···O5A^i^	0.88 (7)	2.06 (7)	2.849 (5)	149 (6)
C7—H7···O1A	0.98	2.55	3.525 (6)	172.
C11—H11A···O3^ii^	0.97	2.45	3.278 (6)	143.
C21B—H21B···O5B^iii^	0.93	2.52	3.369 (9)	151.

Symmetry codes: (i) *x*−1, *y*, *z*; (ii) −*x*+1, *y*−1/2, −*z*+3/2; (iii) *x*, *y*−1, *z*.
